# Improved clinical care and capacity through an integrated electronic patient-reported outcome measure and health record system in inflammatory arthritis

**DOI:** 10.1093/rap/rkaf101

**Published:** 2025-08-29

**Authors:** Antoni Chan, Kathryn Rigler, Nneoma Zabbey, Liz van Rossen, Mustansar Hussain, Andrew Hubbard

**Affiliations:** Department of Rheumatology, Royal Berkshire NHS Foundation Trust, Reading, UK; Digitalisation, Marketing and Entrepreneurship Department, Henley Business School, University of Reading, Reading, UK; Department of Rheumatology, Royal Berkshire NHS Foundation Trust, Reading, UK; Department of Rheumatology, Royal Berkshire NHS Foundation Trust, Reading, UK; Physiotherapy Department, East Kent Hospitals University NHS Foundation Trust, Canterbury, UK; Information Management and Technology Department, Royal Berkshire NHS Foundation Trust, Reading, UK; Information Management and Technology Department, Royal Berkshire NHS Foundation Trust, Reading, UK

**Keywords:** electronic patient-reported outcome measures, inflammatory arthritis, digital technology, remote monitoring, patient-initiated follow-up, personalised care

## Abstract

**Objectives:**

Patient-reported outcome measures (PROMs) are essential for inflammatory arthritis (IA). Collecting electronic PROMs (ePROMs) remotely into electronic patient records (EPRs) enables timely, patient-centred care. This study evaluated the real-world clinical impact, efficiency and feasibility of implementing a fully integrated ePROM system across three IA conditions: rheumatoid arthritis, axial spondyloarthritis and psoriatic arthritis.

**Methods:**

From January 2019 to December 2024, IA patients completed ePROMs remotely at regular intervals or on request by patients or clinicians, with data automatically uploaded to the EPR. Alerts based on predefined thresholds prompted clinical review. Patients with stable disease were transitioned to patient-initiated follow-up. Outcomes include ePROM completion rates, patient engagement, satisfaction, appointment utilisation, time savings and the new-to-follow up (N:FU) ratio. Time saved was estimated by calculating reduced follow-up appointments multiplied by 20-min consultation length. We assessed effectiveness through patient and clinician engagement and satisfaction.

**Results:**

The ePROM completion rates improved from 25% (paper) to 66% (ePROM). A total of 1500 clinic hours per year were saved through reduced follow-ups. The N:FU ratio improved from 1:3.1 in 2019 to 1:2.2 in 2024. Increased capacity enabled shorter waiting times for new and urgent follow-up patient clinic appointments. Longitudinal ePROM trends provided better actionable insight than single time points.

**Conclusion:**

The integrated ePROM system enhanced PROM completion, enabled safe remote monitoring and supported expansion of patient-initiated follow-up. The system improved the efficiency and responsiveness of IA care as well as promoted personalised care.

Key Messages

- Measurement of patient-reported outcome measures (PROMs) are improved using digital solutions.
- Integration of electronic PROMs into EPRs provided clinicians with data for personalised patient management and care.
- A longitudinal study using electronic PROMs provided dynamic remote monitoring that improved clinic scheduling and capacity.

## Introduction

The management of inflammatory arthritis (IA), including RA, axial spondyloarthritis (axSpA) and PsA, requires accurate disease activity monitoring and timely interventions to achieve optimal outcomes [1–3]. Traditional paper-based patient-reported outcome measures (PROMs) often lack completeness and hinder workflow efficiency, limiting their utility in routine practice [4, 5]. As paper-based PROMs are done in person while attending the clinic, this can result in a lack of monitoring if there is a long-time interval between clinic appointments. It is also a time-consuming process in the clinic, as it requires completion, evaluation and transcribing into the patient record.

With the increasing adoption of electronic patient records (EPRs), collecting electronic PROMs (ePROMs) presents an opportunity for advancing patient care in IA [6]. The use of ePROMs enables regular measurements, as opposed to the usual measurement at a single time point in a clinic [7]. Collecting patient data remotely brings the patients’ perspective into routine clinical care without delay. Moreover, connection to EPRs has led to a smooth, integrated and optimised care process [8]. The ePROMs done during the period leading up to the clinic appointment are more reflective of the actual state of a patient’s condition [9]. It also helps to balance patient needs with clinic demand and capacity needs.

We implemented an ePROM system that was integrated into our EPR with the aim of using this as a remote monitoring (asynchronous, data-based review) and virtual follow-up (synchronous consultation via telephone or video) system of patients with IA. The primary aims were to evaluate the pre- and post-implementation PROMs completion rates as well as patient and healthcare professional acceptability. The program was geared towards simplifying the process of PROMs collection, virtual monitoring on the EPR and improving patient outcomes through regular measurements. Patient lists were reviewed regularly using a pre-populated dashboard with the ePROM results. This supported patient-initiated follow-up (PIFU) models to be rapidly implemented on priority pathways, reduce administrative time and improve data quality through digital assessments. We also evaluated the time saved as well as a reduction in wait times from implementing the ePROM system.

## Materials and methods

An assessment of the feasibility of implementing an ePROM system was registered and presented to the Royal Berkshire NHS Foundation Trust Audit and Governance department in 2018 and received approval. As this was a service improvement program, Research Ethics Committee approval was not required. A data protection impact assessment was completed and presented to the Trust Information Governance and received approval in 2018. A business case was submitted in 2018 for the funding and evaluation of the return of investment. This study adhered to the Standards for Quality Improvement Reporting Excellence (SQUIRE) 2.0 guidelines to ensure a structured and transparent presentation of the quality improvement methodology, intervention and outcomes [10]. The SQUIRE framework was used to describe the project’s context, problem identification, intervention design, implementation strategy and evaluation of impact on form completion rates over 6 years.

The intervention involved the phased implementation of an ePROM platform into clinical workflows, including staff training, patient on-boarding procedures and ongoing feedback to teams. PROMs were integrated into care using automated reminders and reviewed during consultations where applicable. AxSpA and PsA data were available from 2019, while RA recruitment began in 2021. The primary outcome was the completion rate of ePROMs, defined as the proportion of recruited patients who completed at least one set of condition-related ePROMs within the reporting year. Data were stratified by disease group and year. Secondary outcomes included the number of patients placed on PIFU, new:follow-up (N:FU) ratio, clinical time saved, wait times and patient/clinician satisfaction.

### NASSS framework for ePROM system implementation

In order to evaluate the ePROM system in real time, the NASSS (non-adoption, abandonment, scale-up, spread, susceptibility) framework was used [11]. This provided a framework for analysing the implementation of healthcare technologies [12]. The framework consists of seven domains: the condition or illness that the technology was intended to address (IA), the technology itself (ePROMs), the value proposition of the technology (time and financial savings), the adopter system (the organization and its staff who would implement and use the technology), the innovation system (the wider context in which the technology is developed and implemented), the patient perspective (evaluation through surveys and feedback) and the ethical and regulatory considerations. The evaluation of the NASSS framework for the implementation of the ePROMs system is shown in Table 1.

**Table 1.** NASSS framework for ePROM system implementation.

1. The illness or health condition	The ePROMs system was intended to improve patient outcomes in chronic disease management of IA, including RA, axSpA and PsA.
2. The technology	The e-PROMs system is a web-based platform that allows patients to complete questionnaires electronically and view their results. The system is integrated with the EPR to ensure that data are shared appropriately with the clinical team.
3. The value proposition	The ePROMs system aimed to improve patient outcomes and experience by enabling patients to monitor their own health and well-being, identify potential issues early and communicate more effectively with their healthcare providers. Through the collection of patient data remotely, it brings the patients’ perspective into routine clinical care without delay and allows for seamless integrated optimised care.
4. The adopter system	Implementing the e-PROMs system required changes to existing workflows and processes within the healthcare organisation. Staff needed to be trained in the use of the system and supported to make the transition from paper-based PROMs to electronic PROMs.
5. The organisation	Implementation of the ePROMs system in the Rheumatology Department of the Royal Berkshire Hospital. The ePROMs application was developed by the Rheumatology Department, Trust IT team and independent technology providers.
6. The wider system with institutional and societal context	Assessment of interoperability across different IT modules and connection to the EPR was undertaken. Evaluation of human factors included patients’ satisfaction and completion rates of ePROMs, interaction with patients, patient consent, patients’ lack of understanding of the process, concerns regarding data safety and lack of equipment. Patient privacy and confidentiality considerations were evaluated.
7. The interaction and mutual adaptation between all these domains over time	Evaluation of the clinical effectiveness and practicality of implementing a fully integrated ePROM system into the hospital EPR in real-world clinic settings, the uptake and acceptability of completing patient outcomes and trend-based ePROMs collated over a period, particularly when considered alongside interventions that were introduced into the clinics. Assessment of the ongoing costs and funding. Time saved in the clinic because of ePROMs was measured.

### Information technology (IT)

**Figure 1.**
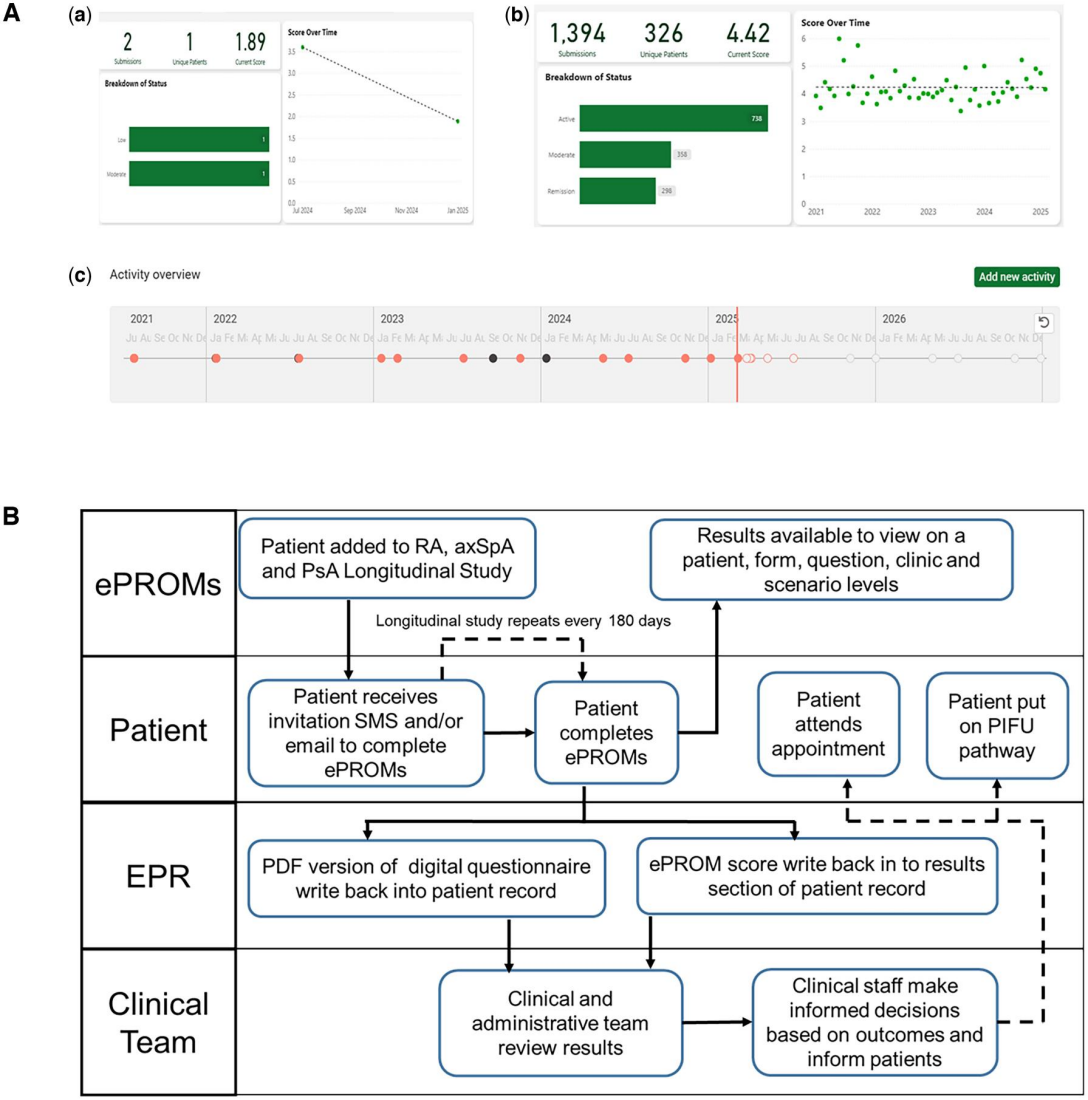
The ePROMs dashboard and digital pathway. **(A)** The ePROMs clinical dashboard showing the BASDAI scores from patients with axSpA from 1 January 2021 to 30 November 2023. **(a)** The clinical status of a patient based on the BASDAI score. **(b)** The clinical status of the axSpA group based on the BASDAI score with remission (<2), moderate (2–4) and active (>4) disease. **(c)** The longitudinal scores of patients from 2021 with regular scores every 180 days and additional ad hoc scores. **(B)** The rheumatology ePROMs digital pathway for remote monitoring of patients with IA. The clinical team included nurses and doctors who reviewed the results daily. The ePROMs system was integrated into the EPR, was interoperable with other devices and was interactive between patients and the clinical team

The ePROMs application was developed by the Rheumatology Department, Trust IT team and independent technology providers (Amplitude and DrDoctor). For the implementation of a digital rheumatology pathway, the following steps were taken. First, digital versions of the rheumatology PROM forms were created. This enabled patients to receive and complete forms ahead of their appointment, at regular intervals and in their own time. Second, integration between the ePROMs system and EPR (Oracle Health Millennium) allowed healthcare professionals and administrative teams to easily view patient results without having to log into multiple systems. Third, a dynamic dashboard was created with breakdowns of disease statuses based on provided scoring cut-offs to help clinical teams make informed decisions on patient care, including PIFU (Fig. 1A). Once ePROMs were completed, results were reviewed by clinical staff via a digital dashboard. Patients with high PROM scores triggered alerts that were integrated into the clinical dashboard. Feedback was provided when clinically indicated. Where appropriate, patients were contacted by phone and a management plan, including an earlier consultation, provided. A standard operating procedure ensured each alert was addressed within 72 h. No structural remuneration currently exists and the system was deployed within existing staff roles and budget for remote monitoring, requiring no additional personnel.

Patients received a web link within Short Messenger Service (SMS) text messages or an e-mail to complete the ePROMs. If a patient did not complete their assessment digitally, prior to the due date, a reminder notification was sent via e-mail and/or an SMS message. The reminder was sent 7 days before it was due. The ePROMs system was designed to work across multiple browsers on a desktop or smartphone to facilitate uptake by both the Trust and patients. There were two views, one for the Trust and one for the patient, allowing each a unique view of the questionnaire and result analysis.

### Patient recruitment

Patients with IA who attended rheumatology clinics at the Royal Berkshire Hospital were recruited into the ePROMs system. Patient educational materials and training were also provided prior to the use of ePROMs. The ePROMs patient education leaflet is shown in Supplementary Fig. S1. Patients were provided structured training materials, including videos and written guides, to support joint self-scoring [13]. This was supported by clinician confirmation during scheduled assessments. Once a diagnosis of IA was made, a treatment plan was created and patients entered the ePROMs system. The alerts from the dashboard were reviewed daily by designated clinical staff (nurses or doctors). The pathway for the ePROMs system is shown in Fig. 1B. The ePROMs sent to IA patients are shown in Table 2. Cut-offs were set for scores and patients with low disease activity (e.g. BASDAI <2, ASDAS <2.1, PSAID-12 <4 and RAPID-3 <3). The selection for PIFU was supported by the ePROMs but not solely based on it. PIFU eligibility was determined through a combination of sustained low disease activity, clinician review, medication stability, patient preference and the absence of red-flag symptoms or disease instability. The lists of patients based on the ePROMs scores were manually reviewed and appointments scheduled based on these results. Patients were sent reminders to complete the questionnaires through SMS or e-mail before each appointment and every 180 days routinely in the longitudinal study group. Patients and clinicians could also send ad hoc forms to patients based on clinical need.

**Table 2.** The ePROMs sent to patients with IA.

PROM questionnaire	RA	axSpA	PsA
Pre-clinic assessment, longitudinal study and ad hoc forms	Routine Assessment of Patient Index Data 3 (RAPID3) Health Assessment Questionnaire (HAQ) Likert Pain Score Tender Joint Count Swollen Joint Count	Bath AS Disease Activity (BASDAI) Bath AS Functional Index (BAFI) Bath AS Global (BASG) Spinal Pain Numerical Rating Scale (NRS) Axial Spondyloarthritis Disease Activity Score (ASDAS)	12-item Psoriatic Arthritis Impact of Disease (PSAID-12) Health Assessment Questionnaire (HAQ) Likert Pain Score Tender Joint Count Swollen Joint Count
Annually	Cardiovascular Risk Score (Q-RISK) Fracture Risk Assessment Tool (FRAX)	Cardiovascular Risk Score (Q-RISK) Fracture Risk Assessment Tool (FRAX)	Cardiovascular Risk Score (Q-RISK) Fracture Risk Assessment Tool (FRAX)
Additional questionnaires sent out dependent on clinical assessment	Hospital Anxiety and Depression Score (HADS) Functional Assessment of Chronic Illness Therapy–Fatigue (FACIT-F) Work Productivity and Activity Impairment (WPAI) Jenkins sleep score Likert general well-being score Likert fatigue score	Hospital Anxiety and Depression Score (HADS) Functional Assessment of Chronic Illness Therapy–Fatigue (FACIT-F) Work Productivity and Activity Impairment (WPAI) Jenkins sleep score Likert general well-being score Likert fatigue score	Dermatology Life Quality Index (DLQI) Hospital Anxiety and Depression Score (HADS) Functional Assessment of Chronic Illness Therapy–Fatigue (FACIT-F) Work Productivity and Activity Impairment (WPAI) Jenkins sleep score Likert general well-being score Likert fatigue score

The forms were sent out as a preclinic assessment, longitudinal study every 180 days and as an ad hoc form. Annual scores are sent out once every 12 months. Additional scores can be sent out by clinicians based on clinical need as preclinic assessment, longitudinal or ad hoc scores.

### Statistical analysis

Chi-squared tests of independence were used to assess differences in ePROM completion status (completed *vs* not completed) across years for each diagnostic group and to compare trends between RA, axSpA and PsA. The dataset consisted of annual time points from 2019 to 2024. The observed frequencies were arranged in a contingency table and the expected frequencies were computed under the assumption of independence. To assess the impact of ePROMs on outpatient follow-up models we analysed changes in the proportion of patients enrolled in the PIFU pathway between year 1 and year 6 of the program. Annual data on total patient volume and PIFU uptake were extracted. A chi-squared test of independence was used to evaluate whether the proportion of patients placed on PIFU varied significantly across years. A *P*-value <0.05 was considered statistically significant. All analyses were conducted using R version 4.3.3 (R Foundation for Statistical Computing, Vienna, Austria).

## Results

Between January 2019 and December 2024, 4082 patients with IA (RA, *n* = 2353; axSpA, *n* = 769; PsA, *n* = 960) were recruited prospectively as they attended their clinic appointments. Once they gave consent online, ePROMs were collected via a patient engagement platform integrated with the hospital EPR. PROMs from the RA, axSpA and PsA bundles were sent to patients before clinic visits, enabling remote completion and longitudinal monitoring of disease activity. The demographics of the patients are shown in Table 3. From January 2019 to December 2024, a total of 40 503 forms were sent to patients with IA (RA, *n* = 16 774; axSpA, *n* = 13 512; PsA, *n* = 10 217).

**Table 3.** The patient demographics in the study.

Demographics	RA	axSpA	PsA
Patients (*n*)	2353	769	960
Age, years, mean (s.d.)	62.4 (14.5)	42.7 (11.2)	52 (7.9)
Sex, male:female, *n*	1.0:0.4	1.7:1.0	0.8:1.0
Disease duration, years, mean (s.d.)	10.7 (9.2)	13.3 (8.3)	17.0 (13.3)
Patients on conventional synthetic DMARDs, *n* (%)	2154 (91.5)	113 (14.8)	857 (89.3)
Patients on biologic DMARDs, *n* (%)	544 (23.1)	286 (37.2)	256 (26.7)
Forms sent (January 2019–December 2024)^a^, *n*	16 774	13 512	10 217
Forms completed (January 2019 to December 2024)^a^, *n*	9367	6508	5093
Disease outcome scores (June–December 2024, mean (s.d.)	RAPID-3, 3.4 (2.5)	BASDAI, 4.5 (2.6)	PSAID-12, 3.8 (2.5)

The patient demographics from the RA, axSpA and PsA cohorts included in the study from 2019 to 2024. The number of patients recruited, age, sex, disease duration, treatment, total number of forms sent and completed from January 2019 to December 2024 and mean (s.d.) disease outcomes score for the 6-month period of June to December 2024 are shown.

a The ePROMs data for RA is for the period 2021–2024.

### ePROMs notification to completion

In 2018, prior to the implementation of ePROMs, the annual clinical notes audit revealed that 25 of 100 (25%) assessed records had documentation of completion of paper-based PROMs. Patient-reported symptoms were not captured at regular intervals and there was no access to unified PROMS data. The audit in 2018 also showed that, on average, it took 8.5 min to complete the paper form from the time of the patient check-in to the time the patient saw the healthcare professional.

**Figure 2.**
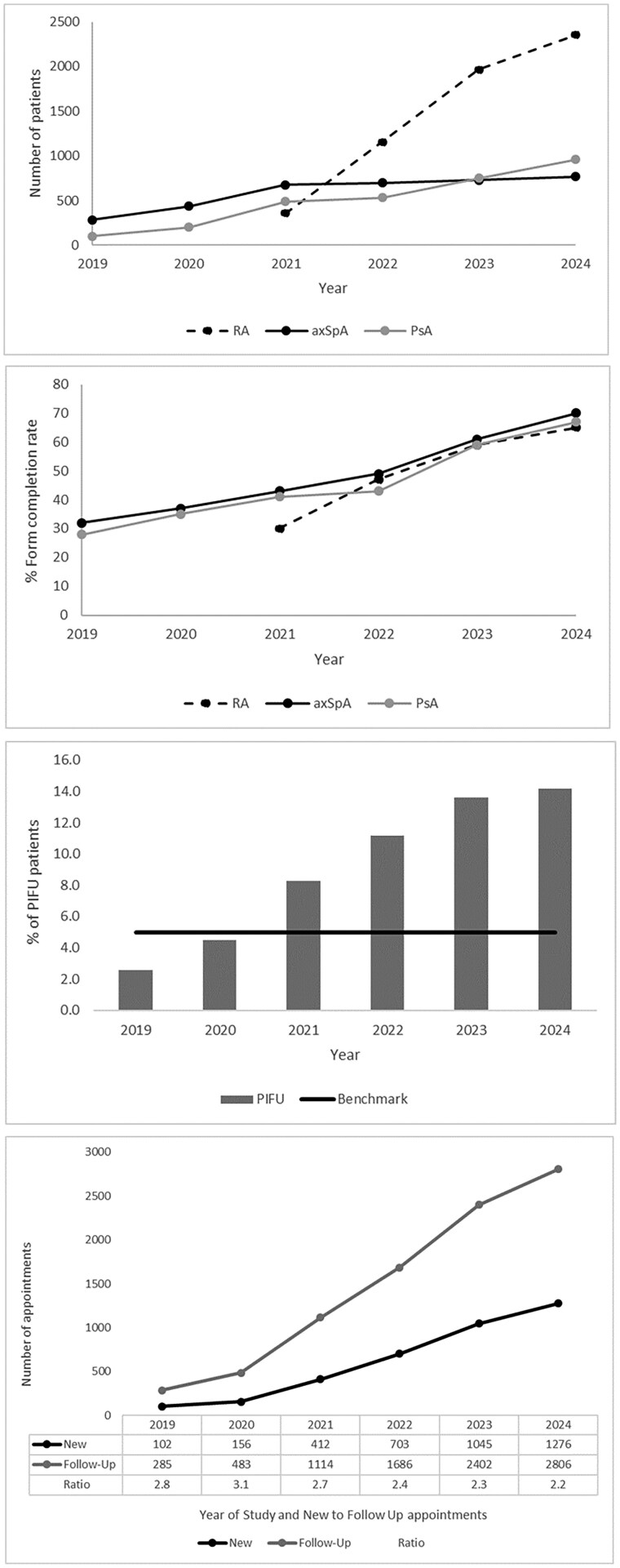
The number of patients recruited, completion of ePROMs, PIFU and N:FU ratio. **(A)** The number of patients with RA, axSpA and PsA recruited into the study from 2019 to 2024. RA patients were only recruited from 2020 onwards. **(B)** The notification to completion rate for IA patients from 2019 to 2024. **(C)** The percentage of patients on PIFU from 2018 to 2024. The horizontal line is the national benchmark for PIFU at 5%. **(D)** The number of new and follow-up appointments and the N:FU ratio for IA patients from 2019 to 2024

The uptake of ePROMs showed notable variations across disease groups and over time. PROM completion rates increased significantly in all three diagnostic groups. In RA, where PROM collection began in 2021, completion rates rose rapidly from 461/1538 (30%) forms in the first year to 4091/6294 (65%) by 2024, reflecting strong integration into clinical pathways. For axSpA, a steady upward trajectory in completion of forms was observed from 2019 [483/1510 (32%)], culminating in a peak completion rate of 1440/2057 (70%) forms in 2024. PsA followed a similar pattern, with marked gains in completion of forms from 2019 [151/541 (28%)] onwards, reaching 1721/2568 (67%) completed forms by 2024. These trends are shown in Fig. 2A, which shows the recruitment of patients with IA from 2019 to 2024. Fig. 2B shows completion rates as percentages of total forms sent, highlighting the relative growth in engagement across the three disease areas.

There were statistically significant differences in ePROM completion rates over time within each diagnostic group. Among patients with RA, completion rates increased markedly between 2021 and 2024 (χ^2^ = 773.03, *P* < 0.0001), indicating substantial year-on-year improvement. For axSpA, ePROM completion also improved significantly from 2019 to 2024 (χ^2^ = 849.38, *P* < 0.0001), although the trend was more gradual compared with RA. In the PsA cohort, a similar pattern emerged, with PROM completion rates rising consistently across the study period (χ^2^ = 684.22, *P* < 0.0001). Notably, while all three groups showed significant improvements, the timing and scale of the changes varied. RA showed the most rapid increase following the introduction of PROMs in 2021, whereas axSpA and PsA demonstrated steadier gains over a longer time frame.

### PIFU

Over the 6-year period, the number of patients on PIFU increased from 10 (2.6%) in year 1 to 580 (14.2%) in year 6 (Fig. 2C). The chi-squared test revealed a statistically significant association between year and PIFU uptake (χ^2^ = 115.54, *P* < 0.0001), indicating a strong upward trend in adoption.

### N:FU ratio

The service started out with an N:FU ratio of 1:2.8 in 2019, which meant for each new patient, there were 2.8 follow-up patients in the clinics over 1 year. This refers to the IA patients enrolled in the ePROMs system. The N:FU ratio rose to 1:3.1 in 2020 during the COVID-19 pandemic, largely due to virtual follow-up consultations due to social distancing. There was a steady trend in the reduction of the N:FU ratio from 1:2.7 in 2021 to 1:2.2 in 2024. The number of appointments and the N:FU ratio is shown in Fig. 2D.

### Clinic time saved

Before the implementation of e-PROMs, follow-up appointments were scheduled every 3–6 months, equivalent to two to four visits per year per patient. With e-PROMs, follow-up frequency was reduced to 12 to 24 months, equivalent to 0.5–1 visit per patient per year. The appointments saved in a year increased from 641 in 2019 to 4694 in 2024. Each follow-up appointment was 20 min long and clinic time saved increased from 214 h in 2019 to 1565 h in 2024.

### New patients seen and wait times

The reduced follow-up frequency resulted in significant clinic time savings, which were repurposed to increase capacity for new patient appointments. Each new appointment was 30 min long and the number of new appointments created per year increased from 427 in 2019 to 3129 in 2024. As capacity for new appointments increased, there was a corresponding reduction in wait times for a first appointment. There was a reduction in wait times of nearly 50% to see new IA patients, from a baseline of 12.1 weeks in 2019 to 6.4 weeks in 2024.

### Patient and clinical healthcare professionals’ feedback

**Figure 3.**
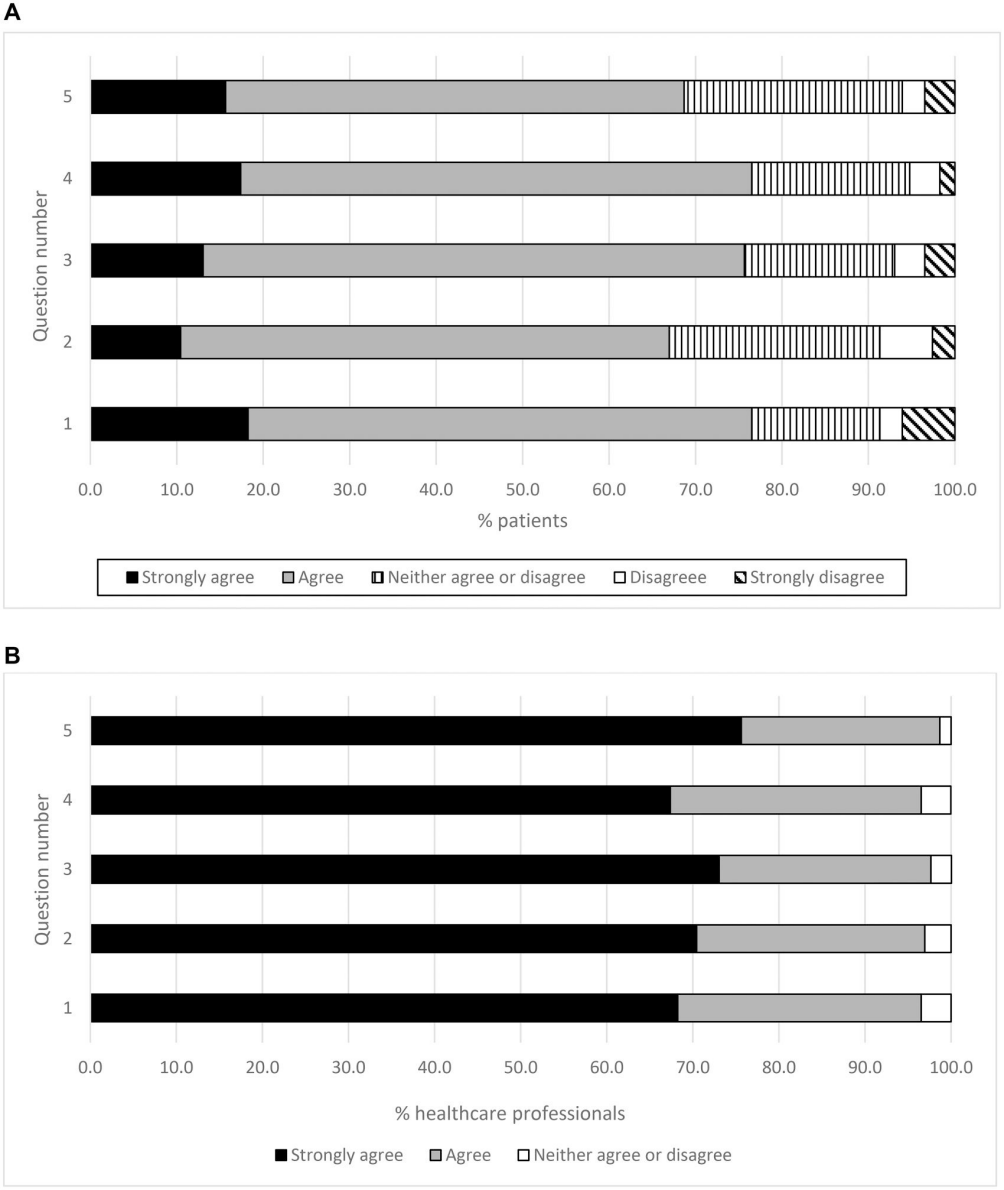
The patient and staff experience and feedback on the use of ePROMs. **(A)** Patient experience and feedback on the use of ePROMs. Questions: (1) It was easy to complete the questionnaire online, (2) I found the questions to be relevant for my care/condition, (3) The guidance on how to complete the form is clear, (4) I understand why I am completing the questionnaire and (5) I prefer using the digital method over other methods of completing forms related to my care. **(B)** Staff experience and feedback on the use of ePROMs. Questions: (1) It was easy to review the questionnaire online, (2) I found having the questionnaire completed online saved me time, (3) I prefer using the digital questionnaire over other methods of data collection, (4) I found using the digital questionnaire help me monitor my patients remotely and (5) I am happy to use these digital questionnaires routinely for my patients

Patients with IA were invited consecutively during the follow-up period to complete a survey in October 2024 on the use of ePROMs. This was completed by 115 patients (Fig. 3A). Patients were asked the following questions (Q): (1) It was easy to review the questionnaire online, (2) I found the questions to be relevant for my care/condition, (3) The guidance on how to complete the form is clear, (4) I understand why I am completing the questionnaire and (5) I prefer using the digital method over other methods of completing forms related to my care. Patients agreed or strongly agreed regarding the ease of use (Q1) in 77% of responses. For relevance (Q2), 67% felt the questions were relevant to their condition. In clarity of guidance (Q3), 76% found the guidance clear. For understanding purpose (Q4), 77% understood why they were completing it. Finally, for preference for digital (Q5), 69% preferred using the digital method. A small proportion of <9% of patients expressed neutrality or dissatisfaction, primarily related to digital literacy or understanding the purpose of the questionnaire. For those who disagreed, this was due to technical difficulties, having multiple forms, frequency of forms sent out, lack of understanding of process, data safety concerns, lack of equipment and patient choice.

Feedback from clinicians was obtained from 30 healthcare professionals in October 2024. These were rheumatologists [*n* = 10 (33%)], specialist nurses [*n* = 12 (40%)], physiotherapists [*n* = 6 (20%)] and pharmacists [*n* = 2 (7%)]. The questions asked were: (1) It was easy to review the questionnaire online, (2) I found having the questionnaire completed online saved me time, (3) I prefer using the digital questionnaire over other methods of data collection, (4) I found using the digital questionnaire helped me monitor my patients remotely and (5) I am happy to use these digital questionnaires routinely for my patients. The feedback was overwhelmingly positive (Fig. 3B). Clinicians agreed or strongly agreed regarding ease of review (Q1) in 97% of the responses. For time saving (Q2), 97% found it saved time. For preference (Q3), 98% preferred digital to other methods. For remote monitoring (Q4), 97% felt it supported remote care. For routine care (Q5), 99% felt they were happy to use ePROMs regularly. For all five questions, <5% of clinicians responded with neutral answers; no responses indicated disagreement.

## Discussion

With the progress in the management of IA, PROMs have been incorporated into the standard of care and treatment guidelines [14]. PROMs have been at the forefront of the assessment of IA, which includes RA, axSpA and PsA [15, 16]. Historically, the clinics followed a strict schedule, with every IA patient being seen every 3–6 months regardless of their condition. Much of the clinic time was spent completing the paper PROMs during the appointment, as it was not always possible to complete them in the waiting room, as outpatient staff were often managing multiple clinics and could not provide patients with PROMs to complete.

Digital ePROMs offer benefits such as automated scoring, data visualisation and improved workflow [17, 18]. The average ePROM completion response in 2024 was 66%, which was above the average reported in the literature of 40–50% [19, 20]. At the start of the study in 2019, the baseline average completion response was lower, at 31%. Other studies have noted that adherence to ePROMs can decrease over time [21, 22]. The attrition rate for ePROMs in longitudinal studies can be mitigated by strategies such as electronic reminders and real-time monitoring [23]. The adherence to completion of ePROMs at the individual patient level over time was monitored on the system. Patients who engage are likely to continue to complete them and patient engagement is key. Including measuring ePROMs as part of the education topics at diagnosis is vital for this.

The utilisation of ePROMs as part of a patient remote monitoring program may support asynchronous (non-live clinician review and triage of ePROM data and alerts) electronic consultations for IA patients [24]. This provides a safety net and surveillance of IA patients on PIFU. This is in line with the NHS Long Term Plan, with digital remote monitoring of patients in the community [25, 26]. Our study highlights the substantial improvement in PIFU uptake across the program years, exceeding the 5% benchmark after year 2 and continuing to rise to year 6. This new model of care is supported by more recent studies that have utilised digital tools for remote monitoring [27, 28].

The N:FU ratio in our study was lower than the national average of 1:4.2, with some hospitals reporting ratios as high as 1:10 [29]. This supports the drive to see the right patient at the right time [30]. As patients were not seen as frequently, education was delivered through digital resources and biannual in-person sessions. Safety monitoring and drug prescriptions were maintained through digital alerts, blood result integration and the clinical advice line. Counselling and interdisciplinary support were available on demand or via patient-initiated contact.

Challenges are present in the implementation of ePROMs, including the need for suitable software solutions and concerns about potential barriers for patients with limited resources [31]. The NASSS framework provided a comprehensive approach to understanding the complexities of implementing digital health technologies, including ePROMs [32]. Studies have demonstrated successful integration of ePROMs into electronic health records, leading to increased completion rates and high acceptability among patients and clinicians [33, 34]. Although we did not measure the psychological burden of repeated confrontation with disease and having to report ePROMs regularly between clinic visits, the patient feedback indicated that it was generally well tolerated. Based on patient feedback, ePROMs were sent out every 180 days rather than more frequently, to avoid questionnaire fatigue and disengagement. As an improvement target, we will need to further study, with continued engagement with patients, the frequency that is best for clinical data that we can use reliably for guiding when clinic appointments or contact with patients is needed. Our study has limitations, as we have not assessed the impact of diverse patient demographics, including ethnicity, socio-economic status, gender, age and comorbidity burden, on the completion rate and adherence to ePROMs [35]. These are areas of future study and will need to be incorporated into the patient education program to overcome barriers to improving ePROM completion rates [36]. Another limitation is the possible response bias from patients and healthcare professionals, as the survey was carried out at a later time point. The generalisability of our findings is most relevant for settings with digital maturity and patient access to online platforms. Scalability will depend on local IT infrastructure, staffing and patient access.

Future plans include scaling this model to other conditions, such as connective tissue diseases, and integrating predictive analytics to detect flares earlier, further optimising personalised care. Our real-world study demonstrates the transformative potential of integrating ePROMs into IA care pathways. By enhancing disease monitoring, reducing in-person visits and empowering patients as active participants in their care, the system significantly improved outcomes and clinic efficiency. This supports a personalised approach in the management of IA patients.

## Supplementary Material

rkaf101_Supplementary_Data

## Data Availability

The data underlying this article will be shared upon reasonable request to the corresponding author.
