# Supplementary material for: Improved clinical care and capacity through an integrated electronic patient-reported outcome measure and health record system in inflammatory arthritis
Source: Rheumatol Adv Pract. 2025 Aug 29;9(4):rkaf101. doi: 10.1093/rap/rkaf101 (PMC12536897; doi:10.1093/rap/rkaf101)
Supplement: rkaf101_Supplementary_Data [file rkaf101_supplementary_data.docx]

**Supplementary Figure S1. The patient information leaflet on ePROMs**

The patient information leaflet on ePROMs that were given to patients at time of recruitment and during educational sessions on management of IA.

**ALT TEXT:** Images showing the document given to patients to inform them about the electronic PROMs system
